# Combined Exposure of Activated Intestinal Epithelial Cells to Nondigestible Oligosaccharides and CpG-ODN Suppresses Th2-Associated CCL22 Release While Enhancing Galectin-9, TGF*β*, and Th1 Polarization

**DOI:** 10.1155/2019/8456829

**Published:** 2019-07-25

**Authors:** Saskia A. Overbeek, Atanaska I. Kostadinova, Martine A. Boks, Simone M. Hayen, Wilco de Jager, Belinda van't land, Leon M. Knippels, Johan Garssen, Linette E. M. Willemsen

**Affiliations:** ^1^Division of Pharmacology, Utrecht Institute for Pharmaceutical Sciences, Faculty of Science, Utrecht University, Utrecht, Netherlands; ^2^Department of Immunology, Danone Nutricia Research B.V., Utrecht, Netherlands; ^3^Molecular Cell Biology and Immunology, VU University Medical Center (VUmc), Amsterdam, Netherlands; ^4^Department of Dermatology and Allergology, University Medical Centre Utrecht, Utrecht, Netherlands; ^5^Department of Pediatric Immunology, Laboratory for Translational Immunology, University Medical Centre Utrecht, Utrecht, Netherlands; ^6^Department of Pediatric Immunology, Wilhelmina Children's Hospital, University Medical Center Utrecht, Utrecht, Netherlands

## Abstract

**Background:**

Short-chain galacto- and long-chain fructo-oligosaccharides (scGOS/lcFOS) and CpG-ODN affect intestinal epithelial cells (IEC). Epithelial IL1*α* may contribute to allergic sensitization via autocrine mediator release affecting dendritic cells (DC). We studied whether IL1*α* contributes to Th2-associated mediator release by activated IEC and IEC/DC cocultures and possible modulation by scGOS/lcFOS±CpG-ODN.

**Methods:**

Solid phase or transwell cultured IEC were preincubated with IL1*α* and/or IFN*γ*/TNF*α* for 6 h. The transwell IEC were also apically exposed to scGOS/lcFOS±CpG-ODN for 6 h, washed, and re-exposed, while cocultured with immature moDC (ccDC) for 48 h. These ccDC were subsequently added to allogeneic naïve T cells (MLR). IEC- and/or DC-derived mediators and T cell cytokines were measured.

**Results:**

IL1*α* tended to enhance IL25 and enhanced IL33 and CCL20 release by IEC, while IL1*α* or TNF*α* or IFN*γ* enhanced CCL22. These were all further increased upon combined exposure of IFN*γ*/TNF*α*±IL1*α* coinciding with increased IL33 secretion in the solid phase culture. In the transwell, IL25 and IL33 remained under detection, while CCL20 and CCL22 were induced by IL1*α* or IFN*γ*/TNF*α*, respectively, and a synergistic increase was observed upon combined exposure of IFN*γ*/TNF*α* and IL1*α*. Furthermore, IFN*γ* was found to enhance galectin-9 secretion, which was more pronounced in IFN*γ*/TNF*α*±IL1*α*-exposed IEC and coincided with TGF*β* increase. Epithelial CpG-ODN exposure further increased CCL20, while reducing CCL22 release by IFN*γ*/TNF*α*/IL1*α*-activated IEC; however, scGOS/lcFOS suppressed both. Combined scGOS/lcFOS and CpG-ODN reduced CCL22, while CCL20 and regulatory galectin-9 and TGF*β* remained high in the supernatant of IFN*γ*/TNF*α*/IL1*α*-activated IEC and the following IEC/DC coculture. ccDC of scGOS/lcFOS- and CpG-ODN-exposed IFN*γ*/TNF*α*/IL1*α*-activated IEC increased IFN*γ*, IL10, TGF*β*, and galectin-9 secretion in the MLR compared to ccDC exposed to control-activated IEC.

**Conclusion:**

IL1*α* enhanced CCL20 and Th2-associated CCL22 release by IFN*γ*/TNF*α*-activated IEC. Combined scGOS/lcFOS and CpG-ODN exposure suppressed CCL22, while maintaining high CCL20, TGF*β*, and galectin-9 concentrations. In addition, ccDC derived from this IEC/DC coculture enhanced Th1 and regulatory mediator secretion mimicking known *in vivo* effects.

## 1. Introduction

The mucosal surface of the gastrointestinal tract is covered by a monolayer of intestinal epithelial cells (IEC). These form a protective barrier between the outside environment and the mucosal immune system of the host, keeping antigenic proteins and bacteria in the lumen, while selectively allowing the transport of nutrients and water [1, 2]. Intestinal antigen-presenting cells, such as dendritic cells (DC), are integral components of the mucosal immune system and control mucosal homeostasis [3]. Intestinal DC can orchestrate durable tolerance to the microbiota and food proteins [3, 4]. IEC are known to support the tolerogenic DC phenotype [5]. However, the process of oral tolerance induction can be disrupted, leading to an inappropriate response towards, for example, a food antigen resulting in food allergies which can provoke gastrointestinal symptoms, atopic dermatitis and/or respiratory symptoms, or even anaphylactic shock [1, 4, 6]. Therefore, strategies to prevent allergy development or to improve oral allergen-specific immunotherapy (OIT) for food allergy are of utmost importance. Dietary intervention using nondigestible oligosaccharides may help to achieve this [7–10].

Recently, it was described that sensitization for inhaled house dust mite in the lung requires IL1*α* release by lung epithelial cells (LEC) resulting in the autocrine induction of Th2-driving IL25, IL33, and TSLP secretion by LEC [11]. These are mediators known to prime Th2-polarizing DC that produce CCL17 and CCL22 and are crucial for allergic sensitization [12]. Besides these Th2-driving cytokines, LEC as well as IEC produce chemokines such as CCL20 that can attract DC and CCL22 which is associated with allergic sensitization [13, 14]. IL1*α* expression was recently also identified in IEC and functions as an alarmin contributing to intestinal inflammation [15]. In the current study, IEC were exposed to IL1*α* to simulate allergen-induced activation. To simulate a coinciding mucosal inflammation, inflammatory mediators IFN*γ* and TNF*α* were used to study the interaction between these types of triggers of epithelial activation and the consequent release of sensitizing mediators.

The prevalence of allergic diseases has increased over the last decades in Westernized countries [6], and there is no effective or curative treatment available. Several reports have proposed the clinical use of dietary nondigestible oligosaccharides (prebiotics) and/or beneficial bacteria (probiotics) or bacterial components in the prevention of atopic diseases such as food allergy [9, 16, 17]. More specifically, dietary supplementation of a specific 9 : 1 mixture of short-chain galacto-oligosaccharides (scGOS) and long-chain fructo-oligosaccharides (lcFOS) and *Bifidobacterium breve* M-16V reduced acute hypersensitivity responses in whey allergic mice [7, 18] and the atopic dermatitis score in infants suffering from IgE-mediated eczema after 12 weeks of treatment [19]. The underlying mechanisms by which scGOS/lcFOS exert these positive effects are unknown. However, this specific oligosaccharide mixture may modulate the immune response by enhancing galectin-9 levels [7], thereby inducing Th1 and regulatory T cell (Treg) polarization in particular in the presence of bacterial-derived CpG DNA or synthetic TLR9 ligand CpG-ODN [7, 20, 21]. Epithelial TLR9 ligation by unmethylated bacterial CpG DNA was found to induce tolerance, thereby maintaining intestinal homeostasis [22]. Both genomic DNA of *Bifidobacterium breve* and synthetic CpG-ODN were shown to enhance Th1 polarization in an IEC-dependent manner, while regulatory IL-10 was also increased [21]. It has been described that galectin-9 can specifically bind IgE, thereby preventing IgE-antigen complex formation leading to reduced degranulation of mast cells and basophils [23]. Moreover, it has been suggested that galectin-9 enhances Treg development and acts synergistically with TGF*β* to enforce induced Treg differentiation and maintenance [23–26]. In addition, increased serum galectin-9 levels upon dietary supplementation with scGOS/lcFOS and *Bifidobacterium breve* M16V were associated with the prevention of asthma-like symptoms in infants affected with atopic dermatitis [7, 19].

Here, we set out to study whether IL1*α* may be a factor contributing to Th2-polarizing cytokine and associated chemokine release by activated IEC. Moreover, it was studied whether this affects DC function and can be modulated by epithelial exposure to scGOS/lcFOS and CpG-ODN.

## 2. Materials and Methods

### 2.1. Culture of Intestinal Epithelial Cells (IEC)

Human colon adenocarcinoma HT29 cells (ATCC, HTB-38; passages 142-148) were cultured in 75 cm^2^ culture flasks (Greiner, Frickenhausen, Germany) in McCoy's 5A medium (Gibco, Life Technologies, Breda, the Netherlands) supplemented with 10% heat-inactivated FCS (Gibco, Life Technologies, Breda, the Netherlands) and penicillin (100 IU/mL)/streptomycin (100 *μ*g/mL) (both Sigma-Aldrich Chemie BV, Zwijndrecht, the Netherlands). HT29 cells were kept in an incubator at 37°C and 5% CO_2_. Medium was refreshed every 2–3 days and cells were passaged once a week.

### 2.2. Solid Phase Model

**Figure 1:**
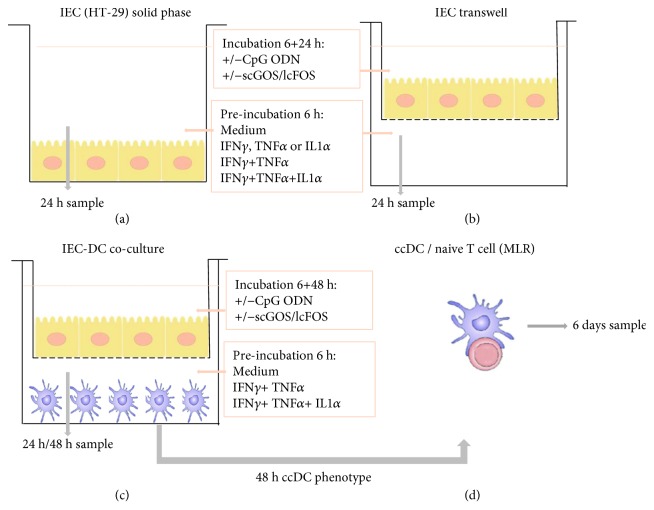
Methods for *in vitro* studies. HT29 cells (IEC) cultured on solid phase plates were preincubated with IFN*γ*, TNF*α*, and IL1*α* (all 10 ng/mL) alone or in combinations for 6 h, washed, and exposed to medium for 24 h (a). IEC cultured on transwell filters (b, c) were basolaterally preincubated with IFN*γ* and TNF*α* in the presence or absence of IL1*α* (all 10 ng/mL) and apically exposed to scGOS/lcFOS±synthetic CpG-ODN for 6 h, washed, and basolaterally exposed to either medium for 24 h (b) or immature DC for 48 h (c), while apically reexposed to medium, scGOS/lcFOS±synthetic CpG-ODN. After 48 h of IEC-DC coculture, ccDC were added to allogeneic naïve T cells for 6 days (MLR) (d) and immune mediators were measured. scGOS/lcFOS: short-chain galacto- and long-chain fructo-oligosaccharides; CpG-ODN: synthetic CpG-ODN type C (TLR9 ligand); imDC: immature DC; ccDC: coculture DC; MLR: mixed lymphocyte reaction.

HT29 cells were cultured in 24-well flat bottom plates (Thermo Fisher Scientific-Nunc, Waltham, USA). After reaching confluence, the cells were incubated with IL1*α* (R&D Systems Europe Ltd., Abingdon, UK), IFN*γ* (Gibco, Life Technologies, Breda, the Netherlands), and/or TNF*α* (Gibco, Life Technologies, Breda, the Netherlands) (all 10 ng/mL) for 6 hours. Subsequently, the cells were washed to remove the cytokines and reexposed to medium for 24 h (Figure 1(a)). After incubation, mediators were measured by Luminex.

### 2.3. Isolation of Monocytes from Healthy Donors

Human peripheral blood mononuclear cells (PBMC) from healthy donors were isolated from buffy coats (Sanquin, Amsterdam, the Netherlands). PBMC were obtained by centrifugation on Ficoll-Paque™ PLUS (GE Healthcare Life Sciences, Uppsala, Sweden; density: 1.077 g/mL). PBMC were collected and washed in PBS (Lonza Westburg BV, Leusden, the Netherlands) + 2% heat-inactivated FCS, followed by hypotonic lysis of erythrocytes with sterile lysis buffer (0.15 M NH_4_Cl, 0.01 M KHCO_3_, and 0.1 mM EDTA pH at 4°C is 7.4, all from Merck, Darmstadt, Germany). After lysis, the PBMC were resuspended in PBS, supplemented with 0.5% BSA (Sigma-Aldrich Chemie BV, Zwijndrecht, the Netherlands) and 2 mM EDTA (pH at 4°C is 7.2). Monocytes were isolated from this PBMC fraction by negative selection using MACS beads and a magnetic cell separator (Monocyte Isolation Kit II, Miltenyi Biotec, Bergisch Gladbach, Germany).

### 2.4. Culture of Monocyte-Derived Dendritic Cells (DC)

Monocytes were cultured at a concentration of 0.75 × 10^6^ cells/mL in a 6-well plate in RPMI 1640 supplemented with 10% heat-inactivated FCS, penicillin (100 U/mL)/streptomycin (100 *μ*g/mL), IL4 (10 ng/mL; ProSpec-Tany TechnoGene Ltd., Ness Ziona, Israel) and GM-CSF (5 ng/mL; ProSpec-Tany TechnoGene Ltd., Ness Ziona, Israel). The cells were kept for 7 days in an incubator at 37°C and 5% CO_2_. At days 2, 3, and 6, 1 mL medium was refreshed. At day 7, the imDC were suitable to use in the transwell IEC-DC coculture assay.

### 2.5. Transwell IEC and IEC-DC Coculture Model

HT29 cells were cultured on transwell inserts (12-well plates, 0.4 *μ*m polyester membrane, Corning, USA). After reaching confluence, the cells were preincubated basolaterally with IFN*γ* and TNF*α* in the presence or absence of IL1*α* (all 10 ng/mL) and apically with medium or 0.5% *w*/*v* of a 9 : 1 mixture of scGOS (Vivinal GOS; Borculo Domo, the Netherlands) and lcFOS (Raftiline HP; BENEO-Orafti, Tienen, Belgium) and/or TLR9 ligand CpG-ODN (M362 type C, 5.0 *μ* m; InvivoGen, San Diego, USA) for 6 hours. Subsequently, the cells were washed and again apically exposed to scGOS/lcFOS±CpG-ODN for 24 hours and basolateral supernatant was collected (Figure 1(b)). To study the IEC-DC crosstalk, immature monocyte-derived dendritic cells (imDC; 0.5 · 10^6^ cells/well) were added basolaterally after 6 h preincubation and cocultured for 48 hours with the IEC (Figure 1(c)), after which the coculture DC (ccDC) were phenotyped and basolateral mediators were measured by Luminex.

### 2.6. Isolation of Naïve CD4^+^ T Cells

Naïve CD4^+^ CD45RO^−^ T cells were purified from the PBMC fraction by negative selection using CD4^+^ T cell isolation kit II (Miltenyi Biotec, Bergisch Gladbach, Germany), together with CD45RO Microbeads (Miltenyi Biotec, Bergisch Gladbach, Germany). Naïve T cells were used as responder T cells in the mixed lymphocyte reaction (MLR).

### 2.7. Mixed Lymphocyte Reaction (MLR)

After 48 hours of coculture with IEC, ccDC were used to stimulate allogeneic naïve CD4^+^ CD45RO^−^ T cells. 10^5^ ccDC were incubated in 24-well flat bottom plates with 10^6^ naïve T cells in IMDM medium (Gibco, Life Technologies, Breda, the Netherlands) supplemented with 10% heat-inactivated FCS, apo-transferrin (20 *μ*g/mL, Sigma-Aldrich Chemie BV, Zwijndrecht, the Netherlands), 2-mercaptoethanol (50 *μ*M, Sigma-Aldrich Chemie BV, Zwijndrecht, the Netherlands), and penicillin (100 U/mL)/streptomycin (100 *μ*g/mL). After 6 days in an incubator at 37°C and 5% CO_2_, the supernatants were harvested and mediators were measured (Figure 1(d)).

### 2.8. Cytokine Production of HT29 Cells, DC, and MLR

Supernatants of HT29 cells (solid phase or transwell) or the IEC-DC coculture or ccDC/naïve T cell MLR were collected as described above. And CCL20, CCL22, IL25, IL33, TGF*β*, and galectin-9 were measured by conducting an in-house developed and validated multiplex immunoassay based on Luminex technology (xMAP, Luminex, Austin, TX USA) as extensively described previously [27]. Acquisition was conducted with the Bio-Rad FlexMAP3D (Bio-Rad Laboratories, Hercules, USA) in combination with xPONENT software version 4.2 (Luminex). Data was analyzed by 5-parametric curve fitting using Bio-Plex Manager software, version 6.1.1 (Bio-Rad).

In the supernatants of the MLR, the cytokines IFN*γ*, IL10, IL13, TGF*β*, and galectin-9 were measured by conducting the aforementioned Luminex.

### 2.9. Statistical Analysis

Data are expressed as mean ± SEM. The statistical significance of the data was analyzed using GraphPad Prism 6.0 software (GraphPad Software, San Diego, CA, USA).

#### 2.9.1. IEC Exposure Experiments

Solid phase: normalized data were analyzed using one-way repeated measures ANOVA followed by Bonferroni's post hoc analysis; transwell: data (normally distributed) were analyzed using two-way repeated measures ANOVA followed by Tukey post hoc analysis.

#### 2.9.2. IEC-DC Coculture Model

Normalized data were analyzed using one-way repeated measures ANOVA followed by Tukey post hoc analysis.

#### 2.9.3. MLR

Data (normally distributed) were analyzed using one-way repeated measures ANOVA followed by Tukey post hoc analysis. Data were considered significant at *p* < 0.05.

## 3. Results

### 3.1. Combined IFN*γ* and TNF*α* Exposure with or without IL1*α* Enhances the Release of Th2-Driving Cytokines and Chemokines by IEC

**Figure 2:**
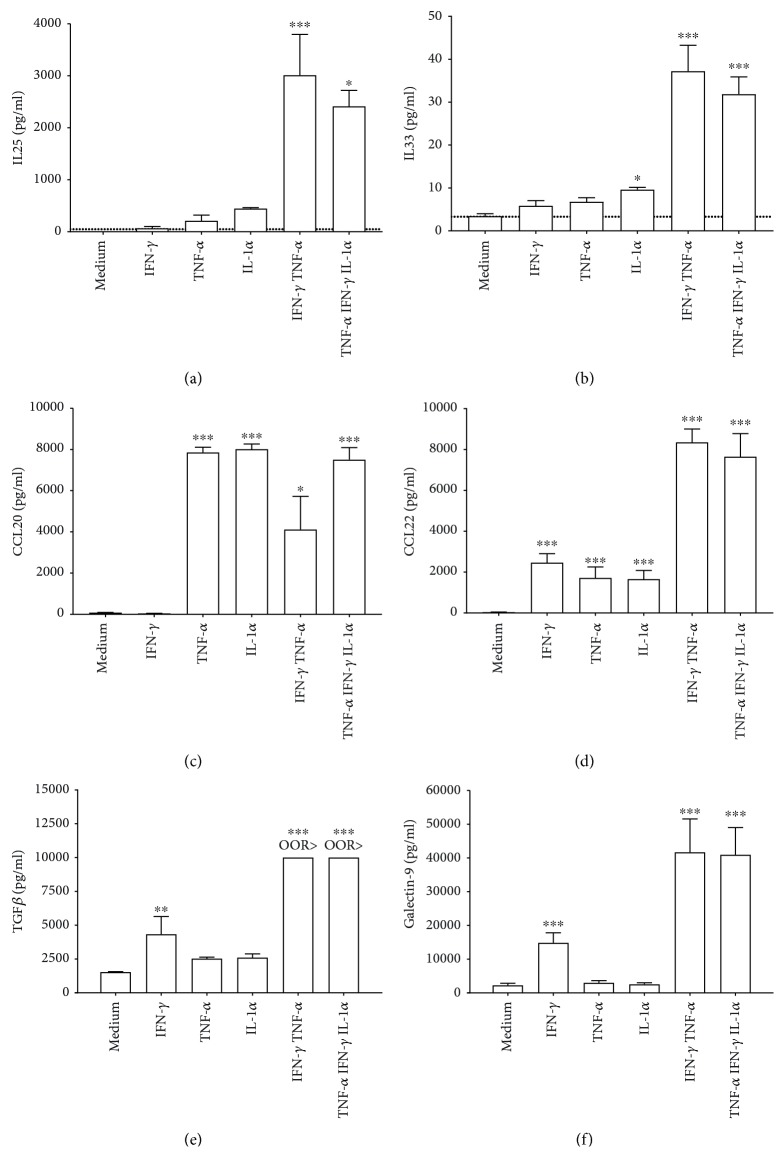
Combined IFN*γ* and TNF*α* exposure with or without IL1*α* enhances the release of Th2-driving cytokines and chemokines by IEC. HT29 cells were cultured in 24-well solid phase plates and preincubated with medium, IFN*γ*, TNF*α*, or IL1*α* (all 10 ng/mL) alone or in combinations for 6 h. Subsequently, the cells were washed and reexposed to medium, after which (a) IL25, (b) IL33, (c) CCL20, (d) CCL22, (e) TGF*β*, and (f) galectin-9 were measured in the supernatants after 24 h; *N* = 3. One-way ANOVA (*p* value interaction for all mediators <0.01), post hoc test Bonferroni; ^∗^ *p* < 0.05; ^∗∗∗^ *p* < 0.001. Ticked line represents the limit of detection (LOD), which is the lowest analyte concentration that can reliably be distinguished from the blank value without consideration of precision and accuracy. OOR> = Out-Of-Range above standard curve.

To study the effect of IEC cytokines on Th2 polarization, solid phase grown HT29 cells were exposed to IL1*α*, TNF*α*, or IFN*γ* alone or in combination (Figure 1(a)). In solid phase cultures, IL1*α* tended to enhance IL25 and enhanced IL33, and IL1*α*, TNF*α*, or IFN*γ* enhanced CCL22 release by HT29 cells. Furthermore, combined exposure to IFN*γ* and TNF*α*±IL1*α* significantly increased the release of IL25, IL33, and CCL22 (Figures 2(a), 2(b), and 2(d)). In addition, TNF*α*, IL1*α*, or IFN*γ* and TNF*α*±IL1*α* activation induced the release of CCL20 (Figure 2(c)). Combined IFN*γ* and TNF*α*±IL1*α* exposure also induced regulatory mediators TGF*β* (Figure 2(e)) and galectin-9 (Figure 2(f)).

Pilot studies showed that established Th2-driving cytokines IL4 and IL13 failed to induce a significant mediator secretion by the HT29 cells, and therefore, these were not used in these studies.

### 3.2. IFN*γ* and TNF*α*±IL1*α* Exposure Provokes Th2-Associated Chemokine CCL22 Release by Transwell IEC, Which Is Suppressed by Incubation with CpG-ODN and scGOS/lcFOS While CCL20 and Regulatory Mediators Remain High

**Figure 3:**
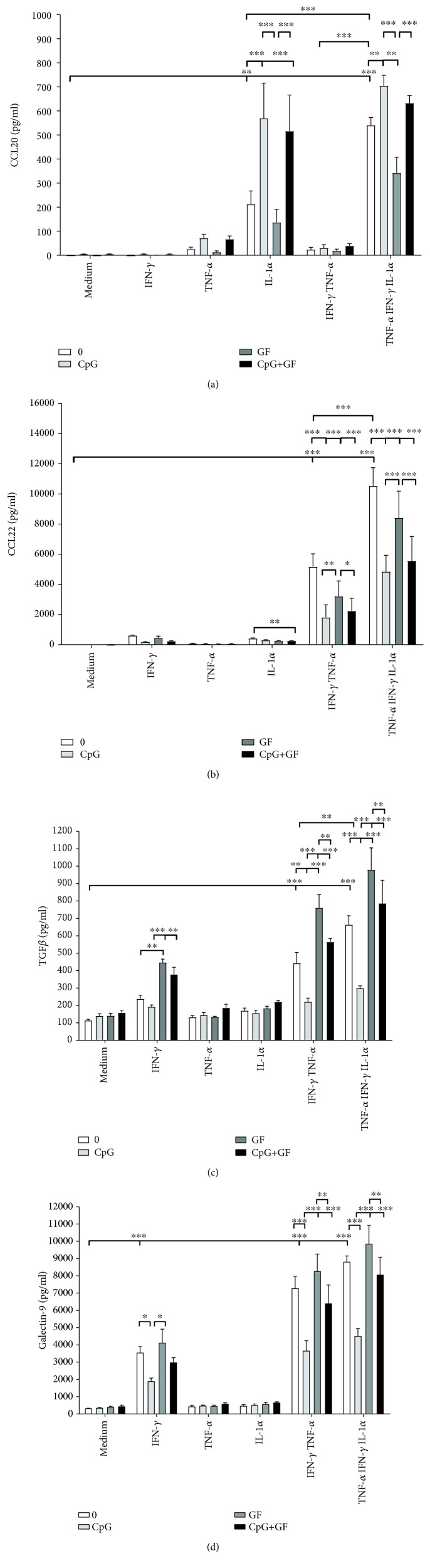
CpG-ODN plus scGOS/lcFOS reduces the release of Th2-associated chemokine CCL22 by IFN*γ*/TNF*α*±IL1*α*-activated IEC, while CCL20 and regulatory mediators TGF*β* and galectin-9 remain high. HT29 cells cultured in transwells were preincubated basolaterally with IFN*γ*, TNF*α*, or IL1*α* (all 10 ng/mL) alone or in combinations and apically exposed to synthetic CpG-ODN (5 *μ*M) in the presence or absence of scGOS/lcFOS (0.5% *w*/*v*) for 6 h. Subsequently, the cells were washed and apically reexposed to CpG-ODN in the presence or absence of scGOS/lcFOS, after which (a) CCL20, (b) CCL22, (c) TGF*β*, and (d) galectin-9 were measured in the basolateral compartment after 24 h; apical exposure to medium (white bars; 0), CpG-ODN (light grey bars; CpG), scGOS/lcFOS (dark grey bars; GF), or CpG-ODN+scGOS/lcFOS (black bars; CpG+GF); *N* = 3. Two-way ANOVA (*p* value interaction for CCL22 <0.05; all other mediators <0.001), post hoc test Tukey; ^∗^ *p* < 0.05; ^∗∗^ *p* < 0.01; and ^∗∗∗^ *p* < 0.001.

To test the effects of CpG-ODN and scGOS/lcFOS exposure to IFN*γ*/TNF*α*±IL1*α*-activated IEC, a transwell model was used (Figure 1(b)). Th2-driving cytokines IL25 and IL33 remained below detection in the HT29 transwell model. However, combined IFN*γ* and TNF*α* activation did induce the release of CCL22 by HT29 cells, which was further enhanced by IL1*α* (Figure 3(b)). CCL20, a strong chemoattractant for DC, was released after activation with IL1*α*. This was further enhanced by IFN*γ* and TNF*α*, although IFN*γ* and TNF*α* activation alone did not lead to a significant release of CCL20 (Figure 3(a)). Apical IEC exposure to scGOS/lcFOS and/or CpG-ODN reduced the release of Th2-associated chemokine CCL22 by IFN*γ*- and TNF*α*±IL1*α*-activated IEC (Figure 3(b)), while CCL20 remained high (Figure 3(a)). Single IFN*γ* exposure and exposure to IFN*γ* in combination with TNF*α*±IL1*α* increased the release of TGF*β* and galectin-9. In addition, while CpG-ODN reduced the concentration of regulatory mediators TGF*β* and galectin-9, TGF*β* increased after scGOS/lcFOS incubation. Furthermore, TGF*β* and galectin-9 remained high when IEC were exposed to the combination of CpG-ODN and scGOS/lcFOS (Figures 3(c) and 3(d)).

### 3.3. ScGOS/lcFOS±CpG-ODN Exposure of IFN*γ*/TNF*α*/IL1*α*-Activated IEC Increases CCL20, TGF*β*, and Galectin-9 Concentrations in Subsequent IEC-DC Coculture While Decreasing CCL22

**Figure 4:**
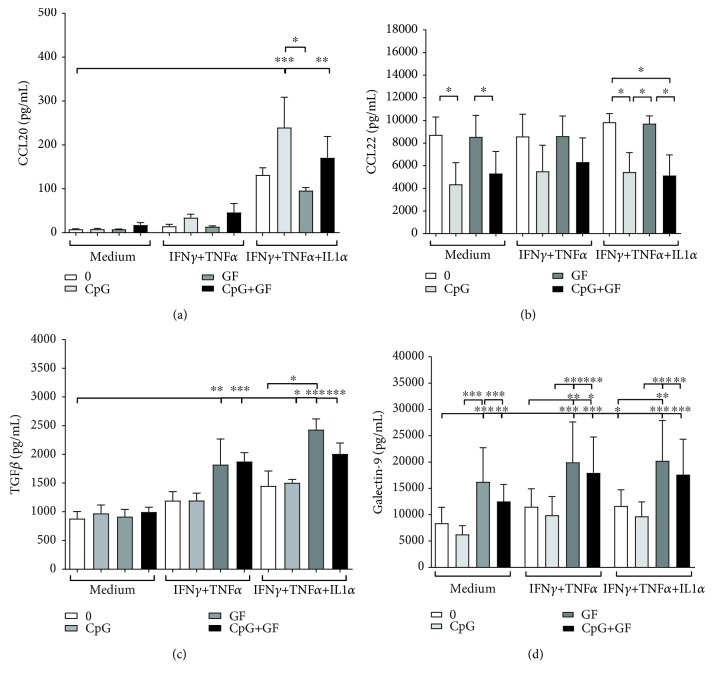
scGOS/lcFOS±CpG-ODN exposure of IFN*γ*/TNF*α*/IL1*α*-activated IEC increases CCL20, TGF*β*, and galectin-9 while decreasing CCL22 in IEC-DC cocultures. After 48 hours of coculture of HT29 cells and imDC, (a) CCL20, (b) CCL22, and (d) galectin-9 were measured in the supernatants. After 24 hours of coculture, (c) TGF*β* was measured in the supernatant. Legend: exposure to medium (white bars; 0), CpG-ODN (light grey bars; CpG), scGOS/lcFOS (dark grey bars; GF), or CpG-ODN+scGOS/lcFOS (black bars; CpG+GF); *N* = 3. One-way ANOVA on normalized data, post hoc test Tukey; ^∗^ *p* < 0.05; ^∗∗^ *p* < 0.01; and ^∗∗∗^ *p* < 0.001.

To investigate whether apical exposure to dietary nondigestible oligosaccharides and bacterial CpG-ODN influences the IEC-DC crosstalk, a coculture experiment with activated IEC and imDC was conducted (Figure 1(c)). After 48 hours of coculture, CCL20, CCL22, TGF*β*, and galectin-9 concentrations were determined in the supernatant of IEC-DC cocultures (Figure 4). CpG-ODN was found to enhance CCL20 in the supernatant of IEC-DC cocultures of IFN*γ*/TNF*α*/IL1*α* IEC-activated IEC cells in the presence or absence of scGOS/lcFOS compared to the intrinsic controls (Figure 4(a)). However, CpG-ODN decreased CCL22 in the supernatant of IEC-DC cocultures of IFN*γ*/TNF*α*/IL1*α* IEC-activated and nonactivated IEC cells in the presence or absence of scGOS/lcFOS compared to the intrinsic controls (Figure 4(b)). Moreover, scGOS/lcFOS was found to enhance galectin-9 in the supernatant of IEC/DC cocultures of activated and nonactivated IEC cells in the presence or absence of CpG-ODN compared to the intrinsic controls. This significant increase in galectin-9 concentrations after 48 hours of coculture was accompanied by a significantly higher release of TGF*β* after 24 hours of IEC-DC coculture (Figures 4(c) and 4(d)).

### 3.4. ccDC Exposed to IFN*γ*/TNF*α*/IL1*α*-Activated IEC Ligated with scGOS/lcFOS and CpG-ODN Significantly Enhance IFN*γ*, IL10, TGF*β*, and Galectin-9 in an Allogeneic MLR

**Figure 5:**
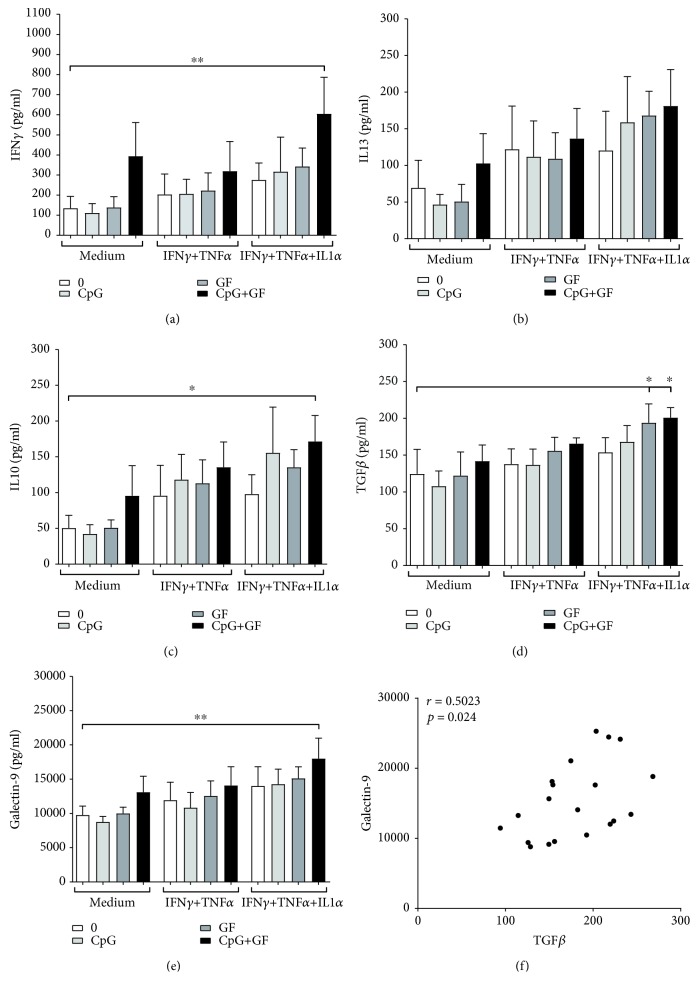
ccDC derived from IEC/DC of IFN*γ*/TNF*α*/IL1*α*-activated IEC ligated with scGOS/lcFOS and CpG-ODN significantly enhance IFN*γ*, IL10, TGF*β*, and galectin-9 in an allogeneic MLR. After 2 days of coculture, ccDC were harvested and washed and ccDC were incubated in a 1 : 10 ratio with allogeneic CD4^+^ naïve T cells. The supernatant was harvested after 6 days of culture and (a) IFN*γ*, (b) IL13, (c) IL10, (d) TGF*β*, and (e) galectin-9 concentrations were measured; *N* = 5. One-way ANOVA, post hoc test Tukey; ^∗^ *p* < 0.05; ^∗∗^ *p* < 0.01; and ^∗∗∗^ *p* < 0.001. (f) Correlation of TGF*β* and galectin-9. Correlation was analyzed using Spearman's correlation test.

The functionality of the ccDC (DC from the IEC-DC coculture) was tested in an MLR with allogeneic naïve CD4^+^ T cells. After two days of IEC-DC coculture, ccDC were harvested and washed (Figure 1(c)). Subsequently, ccDC were incubated with allogeneic CD4^+^ naïve T cells (Figure 1(d)). The supernatant was harvested after 6 days of culture and IFN*γ*, IL10, IL13, TGF*β*, and galectin-9 concentrations were measured. It was observed that IFN*γ*/TNF*α*/IL1*α*-activated IEC did not significantly affect IFN*γ*, IL10, IL13, TGF*β*, or galectin-9 concentrations in the MLR with ccDC originating from the IEC-DC coculture. However, ccDC derived from IEC-DC cocultures with combined scGOS/lcFOS and CpG-ODN exposure of IFN*γ*/TNF*α*/IL1*α*-activated IEC enhanced the IFN*γ*, IL10, TGF*β*, and galectin-9 concentrations compared to ccDC derived from cocultures with medium exposed IEC (Figures 5(a) and 5(c)–5(e)). By contrast, this exposure did not lead to a significant IL13 production (Figure 5(b)). A positive correlation was observed between galectin-9 and TGF*β* in MLR samples derived from ccDC obtained from cocultures of IFN*γ*/TNF*α*/IL1*α*-activated IEC with DC (Figure 5(f)).

## 4. Discussion

In the current study, it was investigated whether IL1*α* may be a factor contributing to Th2-polarizing cytokine and associated chemokine release by activated IEC *in vitro*. Upon exposure to allergens and/or inflammatory insults, IEC may contribute to allergic sensitization since they can produce sensitizing mediators like IL-33, IL-25, and TSLP that are known to contribute to allergic sensitization amongst others via priming of Th2-driving DC. One of the first steps in this cascade may be allergen-induced IL1*α* release by IEC, as was shown for lung epithelial cells, which leads to autocrine stimulation and production of the sensitizing mediators. IL1*α* caused the release of DC chemoattractant CCL20. Moreover, upon exposure to IL1*α* plus inflammatory cytokines, IEC secreted Th2-driving IL25, IL33, and CCL22. CCL22 is also known to be produced by DC that instruct Th2 polarization and allergic sensitization. Interestingly, in the solid phase studies, increased IEC-derived CCL22 release was associated with enhanced IL25 and IL33 secretion, while these mediators were often below detection limits in the transwell studies. CCL22 therefore may be an interesting biomarker when studying factors that activate IEC to release sensitizing mediators. By contrast, IEC are also known to contribute to mucosal homeostasis and tolerance. IEC produce regulatory mediators like galectin-9 and TGF*β* which may be able to modify the priming of the DC and consequent T cell development. Nondigestible oligosaccharides may be able to modulate the mucosal immune response and hereby help to prevent or treat allergies [20]. In the current study, exposure of IEC to scGOS/lcFOS and CpG-ODN (as substitute for bacterial DNA) resulted in decreased CCL22 release by IEC, while supporting the secretion of regulatory mediators like IL-10, TGF*β*, and galectin-9 also by immune cells. In addition, Th1 type IFN*γ* secretion was increased, which is known to dampen Th2 cell activation. This may help to reduce the risk of allergic sensitization for example to food proteins (see Fig.  S2).

In literature, it has been described that IL1*α* released by IEC contributes to intestinal inflammation in mice [15], which may contribute to allergic sensitization or local intestinal inflammation hampering the process of natural oral tolerance induction or the efficacy of OIT. Indeed, in a murine asthma model, it was shown that IL1*α* release by pulmonary epithelial cells induces allergic sensitization to inhaled house dust mite via autocrine release of Th2-driving cytokines IL25, IL33, and TSLP [11]. These cytokines are known to prime DC that produce amongst others CCL22 and induce Th2 cells that secrete IL4, IL5, and IL13 which are crucial for allergic sensitization [12, 28–32]. In addition to the release of Th2-driving cytokines, epithelial cells release chemokines associated with DC chemotaxis and allergic sensitization: CCL20 [33–35] and CCL22 [13, 36]. The current study shows that single exposure to IL1*α*, IFN*γ*, or TNF*α* does not result in significant release of IEC-derived IL-25. However, IL1*α* exposure induced IL33, CCL20, and CCL22 release by IEC, confirming the ability of IL1*α* to promote sensitizing mediator release by IEC. Furthermore, exposure to inflammatory cytokines IFN*γ* or TNF*α* induced the release of CCL22 or CCL20 and CCL22, respectively. However, combined exposure of solid phase grown IEC to inflammatory cytokines IFN*γ* and TNF*α* in the presence or absence of IL1*α* did induce IL25 and IL33 as well as CCL22. In the transwell model of these mediators, only CCL22 was detected, which was further increased by IL1*α* when combined with IFN*γ* and TNF*α*. Interestingly, CCL20 release by IEC was not increased by IFN*γ* and TNF*α* and solely depended on IL1*α* exposure. However, IFN*γ* and TNF*α* further enhanced IL1*α*-induced CCL20 release by IEC. Overall, these data show that combined exposure to IL1*α*, IFN*γ*, and TNF*α* has a strong capacity to activate HT29 cells to release Th2-associated mediators. IL1*α* alone tends to induce epithelial CCL22 release and synergizes with inflammatory mediators IFN*γ* and TNF*α* in activation of epithelial cells. In addition, IL1*α* alone caused CCL20 release, a chemokine known to attract immature DC [37]. This indicates that Th2-polarizing mediators can be released by IEC upon inflammatory insult concomitantly with a DC chemoattractant, which may lead to the development of Th2-driving DC.

Beyond these mediators, IEC also secreted galectin-9 and TGF*β*. Recent reports show that soluble type lectin galectin-9 may be involved in the suppression of allergic symptoms. It was described that galectin-9 can specifically bind IgE, thereby preventing IgE-antigen complex formation leading to reduced degranulation of mast cells and basophils [23, 38]. Moreover, it was suggested that galectin-9 supports Treg development and acts synergistically with TGF*β* to further enforce induced Treg differentiation and maintenance [23–26]. A diet containing scGOS/lcFOS and *Bifidobacterium breve M16*V was shown to partially protect mice from developing food allergic symptoms in association with increased intestinal galectin-9 expression and galectin-9 serum levels [7, 39]. A similar diet also increased galectin-9 levels in serum of infants affected with atopic dermatitis, in association with reduced skin symptom scores [7, 19]. In the current study, IEC secreted galectin-9 after incubation with inflammatory cytokine IFN*γ*. This was further enhanced by TNF*α* in the presence or absence of IL1*α*. In previous studies, epithelial release of galectin-9 was shown to be enhanced by CpG-ODN when IEC were cultured in the presence of activated peripheral blood mononuclear cells and further increased by scGOS/lcFOS [21]. The current study identifies IFN*γ* as an initiator of galectin-9 release by epithelial cells in parallel with regulatory TGF*β* secretion. CpG-ODN ligation in this case suppressed galectin-9 release. However, when combined with scGOS/lcFOS, the suppression of galectin-9 and TGF*β* by CpG-ODN was abrogated. This shows the relevance of studying IEC-derived mediator release which may contribute to the maintenance of mucosal homeostasis.

The modulatory effect of scGOS/lcFOS and/or CpG-ODN on Th2-associated cytokine and chemokine secretion by IFN*γ*/TNF*α*/IL1*α*-activated HT29 cells was also studied. Interestingly, apical exposure of IEC to CpG-ODN reduced the release of Th2-associated chemokine CCL22 in the presence or absence of scGOS/lcFOS, while CCL20 remained high. DC arrive at the site of inflammation via CCL20-CCR6 binding where they are activated and instructed by locally secreted mediators. Upon concomitant epithelial exposure to scGOS/lcFOS, these DC will be exposed to lower levels of CCL22 and high levels of galectin-9 and TGF*β* released by IEC. This may differentially impact the DC phenotype and maturation and consequent instruction of T cell responses upon migration to the lymph nodes. Hence, in addition to reducing mast cell degranulation mediated by galectin-9 [7] and inducing Th1- and Treg-cell polarization [7, 20, 21], IEC exposure to CpG-ODN and scGOS/lcFOS may affect Th2 polarization by decreasing the release of cytokines and chemokines contributing to allergic sensitization by activated IEC, while maintaining high galectin-9 and TGF*β* concentrations. In mice fed a diet containing scGOS/lcFOS and *Bifidobacterium breve* M16V during oral sensitization for hen's egg protein ovalbumin, galectin-9 levels increased while in the lamina propria regulatory T cells were maintained and the Th2 cell frequency was reduced compared to allergic mice fed a control diet [39].

To investigate whether apical exposure of IEC to scGOS/lcFOS and CpG-ODN influences the IEC-DC crosstalk, a coculture experiment with activated IEC and imDC was conducted. IEC pretreated with IFN*γ* and TNF*α* in the presence or absence of IL1*α* did not influence DC maturation. However, scGOS/lcFOS and CpG-ODN ligation of IFN*γ*- and TNF*α*-activated IEC resulted in a significant increase in percentages of more matured CD14^−^DC-SIGN^+^HLA-DR^+^ ccDC. A similar pattern was shown for the percentage of CD14^−^CD40^+^CD80^+^ cells when compared to medium-activated IEC. These effects were lost in the presence of IL1*α* (Fig.  S1). Hence, depending on the type of inflammatory mediators, scGOS/lcFOS and CpG-ODN ligation of IEC may differentially affect the phenotype of the DC exposed to soluble mediators produced by these IEC. In parallel with the studies with activated IEC, also in the IEC-DC cocultures, scGOS/lcFOS reduced CCL20 whereas CpG-ODN in the presence or absence of scGOS/lcFOS enhanced the secretion of CCL20 while reducing CCL22. scGOS/lcFOS enhanced the simultaneous secretion of both galectin-9 and TGF*β* not only in the presence but also in the absence of CpG-ODN either when DC were exposed to control or activated IEC. Therefore, also in the IEC-DC coculture, scGOS/lcFOS and CpG-ODN may modify (tolerogenic) mediator release during inflammatory conditions. This may affect DC maturation and as a consequence its phenotype and function. The functionality of the IEC/DC coculture-derived ccDC was tested in a MLR with allogeneic naïve T cells. It was observed that DC derived from an IEC/DC coculture with IFN*γ*/TNF*α*/IL1*α*-activated IEC did not induce ccDC capable of instructing the release of Th1 cytokine IFN*γ*, Th2 cytokine IL13, and regulatory mediators IL10, TGF*β*, and galectin-9 when compared to medium controls. However, ccDC from IFN*γ*/TNF*α*/IL1*α*-activated IEC/DC cocultures exposed to scGOS/lcFOS and CpG-ODN did enhance the IFN*γ* and IL10 concentrations in this allogeneic MLR compared to medium exposed IEC controls, whereas this exposure did not lead to a significant IL13 production. This may suggest that scGOS/lcFOS and CpG-ODN can skew T cell polarization towards a regulatory Th1 phenotype under these conditions. This may protect against the possible sensitizing capacity of IL1*α* under inflammatory conditions. Indeed, previous *in vitro* studies and studies in mice affected with cow's milk allergy have shown that nondigestible oligosaccharides can support Th1 and regulatory T cell responses [20, 21, 40, 41]. Strikingly, both galectin-9 and TGF*β* concentrations in the MLR using ccDC derived from this coculture were also only increased in the MLR using ccDC from CpG-ODN- and scGOS/lcFOS-exposed IFN*γ*/TNF*α*/IL1*α*-activated HT29 cells. Hence, the current study shows that scGOS/lcFOS adapts the outcome of CpG-ODN exposure on IEC as was shown previously [21], by supporting combined galectin-9 and TGF*β* release. This combined galectin-9 and TGF*β* release did not only occur in the IEC model but also in the IEC/DC coculture and the MLR using ccDC derived from this coculture.

## 5. Conclusions

In conclusion, in these newly developed models in which IL1*α* is used as an additional trigger to provide sensitizing conditions, it has been shown that IL1*α*, TNF*α*, IFN*γ*, and/or a combination of these enhances Th2-associated cytokine and chemokine release by IEC which may contribute to allergic sensitization. In the transwell model, IL1*α* synergized with TNF*α* and IFN*γ* in increasing CCL20 and CCL22 release by IEC. IEC exposure to CpG-ODN and/or dietary scGOS/lcFOS suppressed CCL22 release, while CpG-ODN enhanced CCL20 in the presence or absence of scGOS/lcFOS. Furthermore, IEC exposure to CpG-ODN suppressed galectin-9 and TGF*β* release, while scGOS/lcFOS enhanced these regulatory mediators in the presence or absence of CpG-ODN. DC cocultured with CpG-ODN- and scGOS/lcFOS-exposed IEC instruct Th1 and increase regulatory IL-10, galectin-9, and TGF*β* secretion in a MLR with naïve T cells representing immune priming (see Fig.  S2). This may contribute to the suppression of allergic sensitization.

## Data Availability

The data used to support the findings of this study are included within the article and the supplementary information file.

## Supplementary Materials

Supplementary Materials Figure S1: ScGOS/lcFOS and/or CpG-ODN ligation of IEC and the DC phenotype in IEC-DC crosstalk after initial IEC activation. HT29 cells, cultured on transwell filters, were preincubated basolaterally with IFN*γ* and TNF*α*± IL1*α* (all 10 ng/mL) and apically exposed to synthetic CpG-ODN (5 *μ*M) in the presence or absence of scGOS/lcFOS (0.5% *w*/*v*) for 6 h. Subsequently, the cells were washed and the HT29 cells were again apically exposed to scGOS/lcFOS±CpG-ODN. To study IEC-DC crosstalk, imDC were added basolaterally for 48 hours of coculture (ccDC). Subsequently, the ccDC were collected and incubated with a panel of antibodies (CD14, CD40, CD80, and HLA-DR (all eBioscience, San Diego, CA, USA) and DC-SIGN (R&D Systems Europe Ltd., Abingdon, UK)) and fluorescence was measured by flow cytometry (FACS Canto™ II; BD Biosciences, Franklin Lakes, NJ, USA). The gating technique of fluorescence minus one (FMO) controls was used to interpret the flow cytometry data using FlowLogic software (Inivai Technologies, Mentone, VIC, Australia). Data were normalized by dividing respective percentages by the percentage of the control for every donor. (A) Gating strategy for CD14^−^ population, (B) gating strategy for CD14^−^ CD40^+^ CD80^+^ population, (C) % CD40^+^ CD80^+^ of the living CD14^−^ population, (D) gating strategy for CD14^−^ DC-SIGN^+^ HLA-DR^+^ population, and (E) % DC-SIGN^+^ HLA-DR^+^ of the living CD14^−^ population; exposure to medium (white bars; 0), CpG-ODN (light grey bars; CpG), scGOS/lcFOS (dark grey bars; GF), or CpG-ODN+scGOS/lcFOS (black bars; CpG+GF); *N* = 3. One-way ANOVA on normalized nonparametric data, post hoc test Dunn's; ^∗^ *p* < 0.05. FMO: fluorescence minus one. Figure S2: cartoon of proposed mechanism of action. Structural cells such as intestinal epithelial cells (IEC) may contribute to tolerance induction or allergic sensitization when exposed to allergens and/or inflammatory insults (danger signal). Mucosal allergen exposure may cause release of epithelial-derived autocrine inflammatory IL1*α* and danger signals may provoke release of inflammatory cytokines like TNF*α* and IFN*γ*, produced by mucosal immune cells which may contribute to further increase of epithelial-derived mediator release. IL1*α*-induced release of CCL20 (and CCL22) by IEC may attract immune cells such as DC. Moreover, IL1*α* plus inflammatory cytokines enhances secretion of Th2-driving CCL22 in parallel with IL25 and IL33 secretion by IEC. These mediators may prime Th2-polarizing DC that produce CCL22, leading to Th2 polarization and allergic sensitization (see left site Figure S2). However, other epithelial mediators such as regulatory galectin-9 and TGF*β* may be able to modify the priming of the DC. These mediators may contribute to the production of regulatory mediators such as IL-10, TGF*β*, and galectin-9 by DC and/or T cells and generate Th1 cells to produce IFN*γ* in the presence of regulatory IL-10. Addition of scGOS/lcFOS and bacterial CpG DNA, for example, from bacteria with DNA rich in CpG islands, before or during IEC activation, may decrease CCL22 release by IEC and/or DC (model IEC, model IEC-DC) and support the production of regulatory mediators like IL-10, TGF*β*, and galectin-9 also by DC and/or T cells (model IEC-DC and model ccDC/T-cell), which may modify T cell priming at the inductive site (for example, the mesenteric lymph nodes) and support a more regulatory Th1 response over Th2 polarization (see right site of Figure S2). This may help to reduce the risk of allergic sensitization and allergies to, for example, food proteins.
