# Supplementary figures and images for: Combined Exposure of Activated Intestinal Epithelial Cells to Nondigestible Oligosaccharides and CpG-ODN Suppresses Th2-Associated CCL22 Release While Enhancing Galectin-9, TGFβ, and Th1 Polarization
Source: Mediators Inflamm. 2019 Jul 25;2019:8456829. doi: 10.1155/2019/8456829 (PMC6683774; doi:10.1155/2019/8456829)

Figure S1

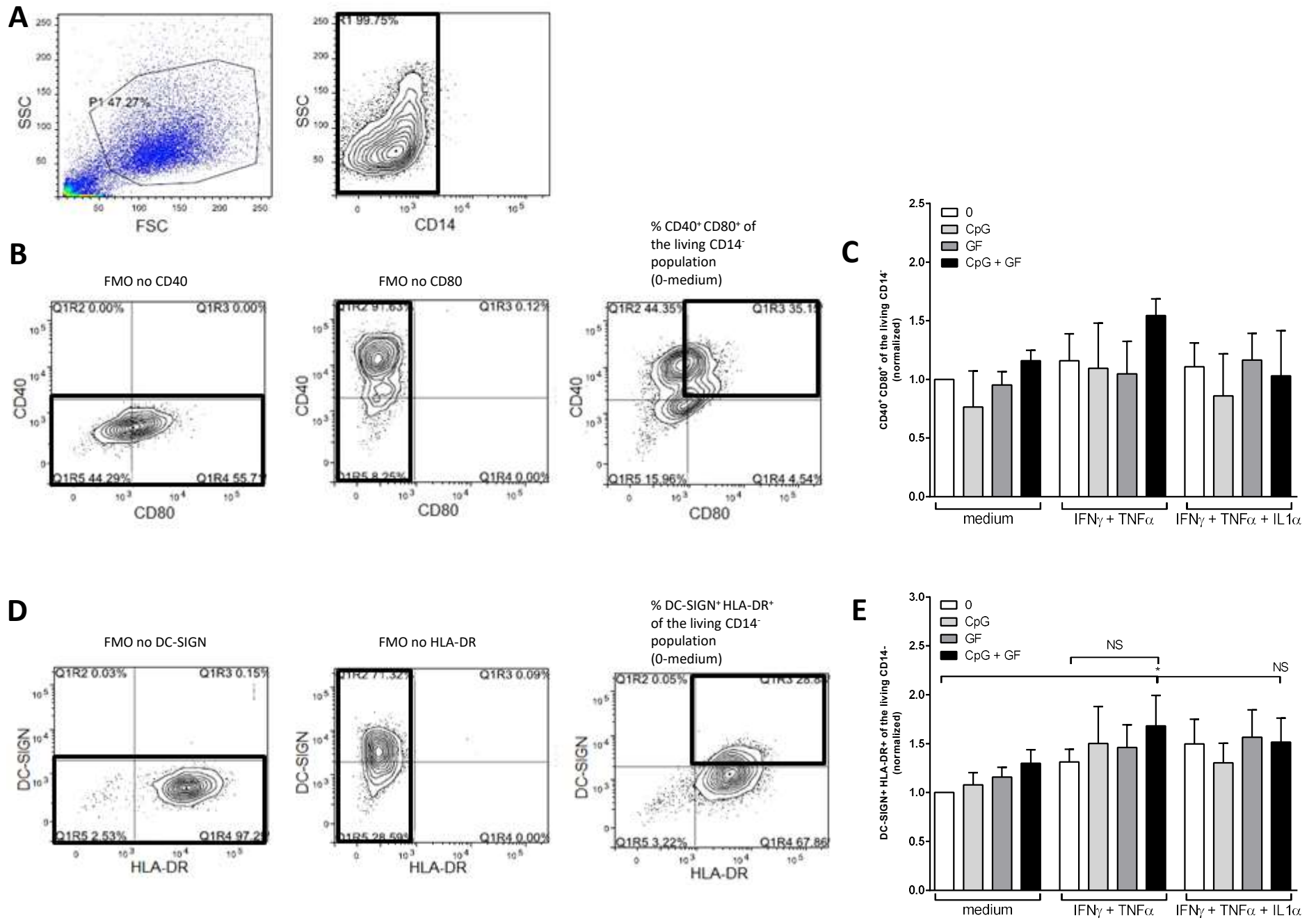

Figure S2

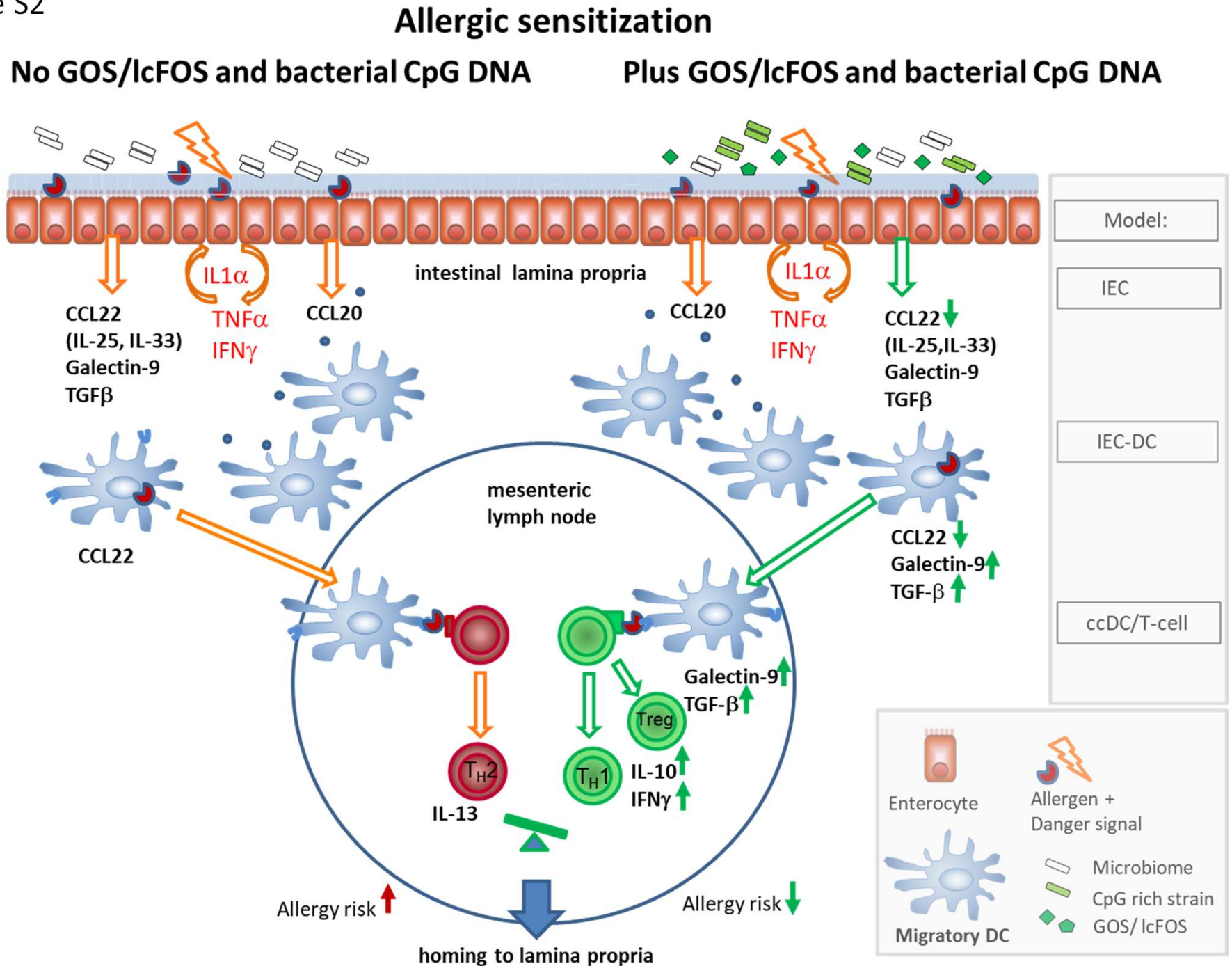

Supplement: Supplementary Materials — Figure S1: ScGOS/lcFOS and/or CpG-ODN ligation of IEC and the DC phenotype in IEC-DC crosstalk after initial IEC activation. HT29 cells, cultured on transwell filters, were preincubated basolaterally with IFNγ and TNFα± IL1α (all 10 ng/mL) and apically exposed to synthetic CpG-ODN (5 μM) in the presence or absence of scGOS/lcFOS (0.5% w/v) for 6 h. Subsequently, the cells were washed and the HT29 cells were again apically exposed to scGOS/lcFOS±CpG-ODN. To study IEC-DC crosstalk, imDC were added basolaterally for 48 hours of coculture (ccDC). Subsequently, the ccDC were collected and incubated with a panel of antibodies (CD14, CD40, CD80, and HLA-DR (all eBioscience, San Diego, CA, USA) and DC-SIGN (R&D Systems Europe Ltd., Abingdon, UK)) and fluorescence was measured by flow cytometry (FACS Canto™ II; BD Biosciences, Franklin Lakes, NJ, USA). The gating technique of fluorescence minus one (FMO) controls was used to interpret the flow cytometry data using FlowLogic software (Inivai Technologies, Mentone, VIC, Australia). Data were normalized by dividing respective percentages by the percentage of the control for every donor. (A) Gating strategy for CD14− population, (B) gating strategy for CD14− CD40+ CD80+ population, (C) % CD40+ CD80+ of the living CD14− population, (D) gating strategy for CD14− DC-SIGN+ HLA-DR+ population, and (E) % DC-SIGN+ HLA-DR+ of the living CD14− population; exposure to medium (white bars; 0), CpG-ODN (light grey bars; CpG), scGOS/lcFOS (dark grey bars; GF), or CpG-ODN+scGOS/lcFOS (black bars; CpG+GF); N = 3. One-way ANOVA on normalized nonparametric data, post hoc test Dunn's; ∗ p < 0.05. FMO: fluorescence minus one. Figure S2: cartoon of proposed mechanism of action. Structural cells such as intestinal epithelial cells (IEC) may contribute to tolerance induction or allergic sensitization when exposed to allergens and/or inflammatory insults (danger signal). Mucosal allergen exposure may cause release of epithelial-derived autocrine inflam [file 8456829.f1.pdf]
